# Supplementary material for: Circulating miR-200 Family and CTCs in Metastatic Breast Cancer before, during, and after a New Line of Systemic Treatment
Source: Int J Mol Sci. 2022 Aug 23;23(17):9535. doi: 10.3390/ijms23179535 (PMC9455626; doi:10.3390/ijms23179535)
Supplement: Supplementary file 1 [file ijms-23-09535-s001.zip › ijms-1885584-supplementary.pdf]

International Journal of
Molecular Sciences

MDPI

Supplementary Material

Circulating miR-200 Family and CTCs in Metastatic Breast Cancer before, during and after a New Line of Systemic Treatment

Table S1 Patient and tumor characteristics. Histology and receptor status refer to the primary tumor of the patient.

				**miR-200a**	**miR-200b**	**miR-200c**	**miR-141**	**miR-429**	**CTC status**
**Total, *N***		47		*p*	*p*	*p*	*p*	*p*	*p*
Age at initial diagnosis (years)	Mean	49.6 *(median 46)*		0.846	0.633	0.7	0.666	0.325	0.930
	Range	(33-77)							
Age at BL (years)	Mean	57.*3 (median 55)*		0.789	0.853	0.4	0.35	0.836	0.73
	Range	(35-89)							
IDC		21	(44.7%)	0.356	0.46	0.574	0.413	0.575	0.351
ILC		8	(17.0%)	0.176	0.483	0.359	0.31	0.269	0.228
Inflammatory		1	(2.1%)	0.461	0.543	0.604	0.371	0.327	0.375
Unknown		17	(36.2%)						
ER status				0.416	0.731	0.551	0.819	0.524	0.438
	Positive	38	(80.8%)						
	Negative	7	(14.9%)						
	Unknown	2	(4.3%)						
PR status				0.409	0.708	0.381	0.895	0.589	0.199
	Positive	32	(68.1%)						
	Negative	11	(23.4%)						
	Unknown	4	(8.5%)						
HER2 status				0.397	0.144	0.276	0.13	0.241	0.241
	Positive	5	(10.6%)						
	Negative	33	(70.2%)						
	Unknown	9	(19.1%)						
Overall survival (months)	Median	21							
	Range	(3-84)							
Progression-free survival (months)	Median	5							
	Range	(1-47)							
Distant metastasis at initial diagnosis				0.637	0.76	0.597	0.742	0.476	0.321
	M0	29	(61.7%)						
	M1	10	(21.3%)						
	Unknown	8	(17.0%)						

Local metastasis				0.026*	0.049*	0.094	0.742	0.476	0.23
	Yes	23	(48.9%)						
	No	24	(51.1%)						
Visceral metastasis				0.837	0.513	0.704	0.272	0.545	0.766
	Yes	25	(53.2%)						
	No	22	(46.8%)						
Bone metastasis				0.003*	0.031*	0.030*	0.037*	0.034*	0.072
	Yes	37	(78.7%)						
	No	10	(21.3%)						
Chemotherapy				0.174	0.135	0.221	0.365	0.115	0.880
	Yes	45	(95.7%)						
	No	2	(4.3%)						
Endocrine therapy				0.283	0.552	0.568	0.657	0.977	0.902
	Yes	35	(74.5%)						
	No	12	(25.5%)						

BL – baseline, IDC – invasive ductal carcinoma, ILC – invasive lobular carcinoma, ER – estrogen receptor, PR – progesterone receptor, HER2 – human epidermal growth factor receptor 2, CTC – circulating tumor cells, *p* – statistical p-value

Table S2 miRNA detection rates of the analyzed miRNA panel across measurements in time

	miRNA$_{BL}$			miRNA$_{1c}$			miRNA$_{PD}$		
miRNA	*N*	mean	SD	*N*	mean	SD	*N*	mean	SD
miR 200a	47	32.52	2.09	47	33.70	2.05	46	32.06	2.47
miR 200b	47	33.22	2.30	45	34.54	2.23	46	32.62	2.65
miR 200c	47	33.72	2.13	47	34.37	2.00	46	32.84	2.42
miR 141	47	33.60	2.82	46	35.57	2.75	46	33.19	3.01
miR 429	47	36.02	2.58	43	37.37	2.74	46	35.39	3.09
miR 375	47	29.69	2.24	47	30.30	2.31	46	29.05	2.75
miR 210	47	44651	1.18	47	31.69	1.68	46	30.33	1.58
miR 365	47	31.46	1.35	47	31.92	1.88	46	30.88	1.58
miR 1260	47	31.02	1.76	47	30.97	1.69	46	30.70	1.50
miR 16	47	23.56	1.26	47	23.74	1.64	46	22.89	1.05
miR 24	47	28.97	1.21	47	28.97	1.21	46	28.34	1.19
miR 29a	47	27.93	0.89	47	28.33	1.07	46	27.42	1.12
miR 138	40	37.98	1.74	35	37.88	1.56	42	37.41	1.38

Note. Cp – crossing point value, miRNA$_{BL}$ – measurement at baseline, miRNA$_{1c}$ – measurement after 1 completed cycle of a new line of systemic therapy, miRNA$_{PD}$ – measurement at progression of disease, *N* – sample size, mean – arithmetic mean, SD – standard deviation

Table S3 Linear regression analyzing miRNA expression and CTC status in relation to relevant clinical characteristics.

	miR 200a		miR 200b		miR 200c		miR 141		miR 429		CTC status	
	Estimate	p-value	Estimate	p-value	Estimate	p-value	Estimate	p-value	Estimate	p-value	Estimate	p-value
Age at initial diagnosis	-0.0	0.846	-0.01	0.633	0.01	0.7	0.02	0.666	-0.03	0.325	-0.0	0.93
Age at BL	0.01	0.789	-0.01	0.853	0.021	0.4	0.03	0.35	0.01	0.836	-0.0	0.73
IDC	0.57	0.356	0.51	0.46	0.36	0.574	0.68	0.413	0.43	0.575	-0.14	0.351
ILC	-1.10	0.176	-0.63	0.483	-0.77	0.359	-1.12	0.31	-1.12	0.269	0.24	0.228
Other	-1.58	0.461	-1.44	0.543	-1.14	0.604	-2.58	0.371	-2.59	0.327	0.46	0.375
ER status	-0.71	0.416	-0.33	0.731	-0.53	0.551	0.27	0.819	0.67	0.524	-0.16	0.438
PR status	-0.60	0.409	-0.30	0.708	-0.67	0.381	0.13	0.895	0.48	0.589	-0.23	0.199
HER2 status	-0.83	0.397	-1.62	0.144	-1.17	0.276	-2.13	0.13	-1.36	0.241	-1.36	0.241
Distant metastasis at initial diagnosis	-0.37	0.637	0.27	0.76	0.43	0.597	-0.35	0.742	-0.67	0.476	-0.19	0.321
Local metastasis	1.36	0.026	1.33	0.049	1.06	0.0936	1.15	0.168	1.33	0.081	-0.18	0.23
Visceral metastasis	-0.13	0.837	-0.45	0.513	-0.24	0.704	-0.93	0.272	-0.47	0.545	0.05	0.766
Bone metastasis	-2.12	0.003	-1.75	0.031	-1.63	0.030	-2.08	0.037	-1.93	0.0341	0.32	0.072
Chemotherapy	2.06	0.174	2.49	0.135	1.90	0.221	1.87	0.365	2.95	0.115	0.06	0.880
Endocrine therapy	-0.74	0.283	-0.45	0.552	-0.40	0.568	-0.41	0.657	-0.02	0.977	0.02	0.902
Bevacizumab	1.74	0.078	1.41	0.199	1.29	0.204	1.02	0.453	1.14	0.357	-0.17	0.477
CTC status	-2.03	<0.001	-2.23	<0.001	-2.02	<0.001	-3.10	<0.001	-2.80	<0.001		

CTC – circulating tumor cells, CTC_{BL}- measurement at baseline, IDC – invasive ductal carcinoma, ILC – invasive lobular carcinoma, ER – estrogen receptor, PR – progesterone receptor, HER2 – human epidermal growth factor receptor 2, Estimate - logistic regression coefficient, p - statistical p-value

Table S4 Results of univariable logistic regression analysis of miRNA expression as a predictor for CTC status

	CTCBL		CTC1C		CTCPD	
miRNA	Estimate	p	Estimate	p	Estimate	p
miR-200a	-0.60	0.003	-0.88	0.001	-0.43	0.008
miR-200b	-0.55	0.003	-0.60	0.004	-0.33	0.016
miR-200c	-0.55	0.003	-0.64	0.004	-0.34	0.021
miR-141	-0.54	0.001	-0.52	0.003	-0.28	0.016
miR-429	-0.58	0.001	-0.51	0.004	-0.29	0.012

CTC – circulating tumor cells, CTCBL – CTC status at baseline, CTC1C – CTC status after 1 completed cycle of a new line of systemic therapy, CTCPD – CTC status at disease progression, p – statistical p-value

Table S5 Results of cox proportional hazard model between OS and miRNA expression. HR calculated as the ratio of the probability of progression of the disease of miRNA high-level group to that of miRNA low-level group.

	CTC_{BL}		CTC_{1C}		CTC_{PD}	
miRNAs	HR (95% CI for HR)	*p*	HR (95% CI for HR)	*p*	HR (95% CI for HR)	*p*
miR 200a	2.59 (1.28-5.21)	0.008	3.12 (1.54-6.31)	0.002	1.91 (0.95-3.85)	0.072
miR 200b	2.59 (1.28-5.21)	0.008	3.05 (1.50-6.22)	0.002	1.62 (0.81-3.24)	0.176
miR 200c	2.54 (1.26-5.12)	0.009	3.69 (1.79-7.61)	<0.001	1.78 (0.89-3.59)	0.106
miR 141	2.50 (1.24-5.04)	0.011	4.27 (2.04-8.91)	0.000	1.70 (0.84-3.44)	0.137
miR 429	2.73 (1.35-5.51)	0.005	3.09 (1.47-6.47)	0.003	2.12 (1.05-4.30)	0.036
CTC status	2.07 (1.12-3.85)	0.021	2.14 (1.16-3.97)	0.015	2.58 (1.38-4.84)	0.003
Age	0.99 (0.97 -1.02)	0.544	0.99 (0.97-1.02)	0.552	0.98 (0.96-1.01)	0.120
M	1.05 (0.37-2.96)	0.930	1.04 (0.37-2.95)	0.937	0.96 (0.36-2.58)	0.936
Local	0.56 (0.30-1.05)	0.071	0.58 (0.31-1.07)	0.080	0.72 (0.39-1.35)	0.309
Visceral	1.49 (0.80-2.76)	0.206	1.53 (0.83-2.84)	0.176	1.10 (0.59-2.06)	0.764
Bone	2.53 (1.14-5.62)	0.023	2.54 (1.14-5.64)	0.022	2.11 (0.93-4.79)	0.074

OS – overall survival, CTC – circulating tumor cells, CTC_{BL} – measurement at baseline, CTC_{1C} – measurement after 1 completed cycle of a new line of systemic therapy, CTC_{PD} – measurement at progression of disease, Age – age at study entry, M – metastatic status at initial diagnosis, Local/Visceral/Bone – location of metastasis, HR – hazard ratio, CI – confidence interval, *p* – statistical *p*-value

Table S6 Results of cox proportional hazard model between PFS and miRNA expression. HR calculated as the ratio of the probability of progression of the disease of miRNA high-level group to that of miRNA low-level group.

	CTC_{BL}		CTC_{1C}	
miRNAs	HR (95% CI for HR)	*p*	HR (95% CI for HR)	*p*
miR 200a	1.49 (0.66-3.39)	0.340	2.25 (1.05-4.82)	0.037
miR 200b	1.49 (0.66-3.39)	0.340	2.16 (1.00-4.66)	0.049
miR 200c	1.84 (0.84-4.07)	0.129	2.81 (1.33-5.93)	0.007
miR 141	1.62 (0.74-3.55)	0.233	2.74 (1.25-6.01)	0.012
miR 429	1.43 (0.63-3.26)	0.390	2.50 (1.11-5.65)	0.028
CTC status	0.80 (0.40-1.57)	0.514	1.63 (0.82-3.25)	0.165
Age	1.00 (0.97-1.03)	0.864	1.01 (0.98-1.05)	0.367
M	1.30 (0.57-2.98)	0.535	1.10 (0.48-2.51)	0.819
Local	0.74 (0.37-1.46)	0.382	0.43 (0.21-0.89)	0.023
Visceral	1.00 (0.51-1.95)	0.99	1.31 (0.66-2.58)	0.440
Bone	0.90 (1.08-7.80)	0.035	2.72 (1.08-6.85)	0.033

PFS – progression-free survival, CTC – circulating tumor cells, CTC_{BL} – measurement at baseline, CTC_{1C} – measurement after 1 competed cycle of a new line of systemic therapy, Age – age at study entry, M – metastatic status at initial diagnosis, Local/Visceral/Bone – location of metastasis, HR – hazard ratio, CI – confidence interval, *p* – statistical *p*-value
